# The Combined Effects of Sublingual Immunotherapy and Lactobacillus acidophilus-Producing Extract on Cedar Pollinosis Symptoms

**DOI:** 10.7759/cureus.41374

**Published:** 2023-07-04

**Authors:** Ito Hirobumi, Yasuhiro Sasuga

**Affiliations:** 1 Otolaryngology, Ito ENT Clinic, Funabashi, JPN; 2 Laboratory, B&S Corporation Hachioji Research and Development Center, Tokyo, JPN

**Keywords:** fast onset of effects, food ingredients, combination effect, salvage therapy, ineffective cases, lactobacillus acidophilus produced extract (lex), sublingual immunotherapy (slit), pollinosis, japanese cedar pollen, seasonal allergic rhinitis

## Abstract

Introduction: Sublingual immunotherapy (SLIT), in which standardized cedar pollen extract solution is administered, has been used to treat cedar pollinosis, but SLIT is problematic because it takes a long time to become effective, and some cases are ineffective even after long-term treatment. It has also been reported that lactobacillus acidophilus extract (LEX), a food-derived ingredient, alleviates various allergic symptoms. This study examined the usefulness of LEX as a treatment for cedar pollinosis in comparison with SLIT. We also examined whether the combined use of SLIT and LEX could have an early-onset of therapeutic effect on cedar pollinosis. We also examined the usefulness of LEX as a salvage therapy for patients who failed to respond to SLIT.

Subjects and Methods: Fifteen patients with cedar pollinosis were divided into three groups. The three groups were: three patients in the standardized cedar pollen extract group (S group), seven patients in the lactobacillus-producing extract group (L group), and five patients in the combination group of standardized cedar pollen extract and lactobacillus-producing extract (SL group). The subjects were treated for three years, corresponding to the three scattering seasons of cedar pollen, and observed according to the evaluation items. The evaluation items were severity score based on examination findings, subjective symptom score (QOL score) based on the Japanese Standard QOL Questionnaire for Allergic Rhinitis (JRQLQ No. 1) questionnaire, nonspecific IgE level measurement by blood test, and cedar pollen-specific IgE level measurement.

Results: After three years of observation, there were no significant differences in severity score and nonspecific IgE levels among the three groups, while QOL score decreased significantly between the first and third years of treatment in the L group. Cedar pollen-specific IgE levels in the S and SL groups showed an increase in the first year and a gradual decrease in the second and third years of treatment compared to the pre-treatment period. In group L, no increase was observed in the first year, and a significant decrease was observed in the second and third years during the cedar pollen dispersal period.

Conclusions: The results of severity and quality of life scores indicated that it took three years of treatment for the S and SL groups to show efficacy, while the L group showed improvement in quality of life scores and cedar pollen-specific IgE levels from the first year, suggesting that LEX is useful as a treatment for cedar pollinosis. The efficacy of combination therapy with SLIT and LEX was not clear, but since the effect of LEX was observed from the early stage of treatment, it was thought that the combination therapy with LEX intake from the early stage of treatment may be effective in reducing the incidence of ineffective cases. The combination therapy of SLIT and LEX may also be useful as a salvage therapy.

## Introduction

In Japan, cedar forests cover 18% of the nation's forest area and are estimated to be responsible for about 70% of pollen allergy cases. When cedar pollen antigens (Cry j 1 and Cry j 2) are recognized by macrophages in the nasal cavity, antigen information is transmitted to T cells and B cells, which produce cedar-specific IgE antibodies and sensitize them. When cedar pollen antigens bind to sensitized IgE antibodies, mast cells are activated and release chemical messengers such as histamine and leukotrienes, resulting in the appearance of allergic rhinitis symptoms caused by cedar pollinosis. Cedar pollinosis in Japan is on the increase. In a Japanese nationwide epidemiological survey, the prevalence of cedar pollinosis exceeded about 38%, and a decrease in quality of life (QOL) and labor productivity due to cedar pollinosis have been reported [1]. Hay fever patients tend to be young to middle-aged. Symptoms include not only sneezing, nasal discharge, and nasal obstruction, but also fatigue and decreased ability to concentrate, resulting in decreased daytime performance, which has a significant impact on working and schooling in the prime of life. Therefore, treatment of hay fever patients should not only suppress symptoms, but also take into consideration the improvement of QOL in the workplace and school life [2].

Sublingual immunotherapy (SLIT) is a promising curative therapy, which induces regulatory T cells to induce Th1-type immune responses and alleviate Th2-type immune responses. It is also thought to have a mechanism of action such as inducing allergen-specific IgG4 antibodies, which are antibodies that block cedar pollen antigens, in the body [3]. The use of various food-derived ingredients has been investigated for the purpose of improving QOL and treatment of cedar pollinosis. Problems to be solved with SLIT include the long treatment period, the existence of invalid cases, and the difficulty in predicting treatment efficacy [4]. Probiotics such as lactic acid bacteria are one such example, and their effectiveness in reducing allergic symptoms including cedar pollinosis has been reported [5-7]. In addition, sterilized organisms and bacterial metabolites have been reported to be effective as postbiotic materials [8,9]. Lactobacillus fermentation extract (hereinafter referred to as LEX), which is obtained by fermenting soy milk with multiple strains of lactic acid bacteria, has been reported to have anti-colorectal adenocarcinoma effects [10], intestinal immunostimulating effects [11], effects on colon polyps [12], induce changes in the intestinal microflora, improve the intestinal environment improvement [13], etc. have been reported.

There are reports [3] that the usefulness of SLIT and the intake of lactic acid bacteria and components such as probiotics and postbiotics are effective against cedar pollinosis by bringing about improvement of the intestinal environment. However, there are no studies within the scope of the author's search that evaluated the combination therapy of SLIT and LEX or the usefulness of LEX as a salvage therapy for SLIT ineffective cases. This study focused on postbiotic ingredients derived from food for the purpose of establishing early efficacy of combination therapy with SLIT or salvage therapy for invalid cases, and conducted a preliminary study on the use of LEX. The evidence for the immunomodulatory effects of postbiotic ingredients in humans is still limited, and this study is reported here as a potential source of evidence.

The abstract of this paper was presented at the 83rd Annual Meeting and Conference of the Clinical Society of Otolaryngology, June 26-27, 2021, Sapporo, Japan.

## Materials and methods

This study is a quasi-experimental study that followed three groups of patients: those who received SLIT medication alone, those who received LEX alone, and those who received SLIT medication and LEX for three years, from 2016 to 2018. The subjects were patients who visited our clinic in 2015 and were diagnosed with cedar pollinosis, had a history of cedar pollen symptoms during the cedar pollen dispersal period in 2015, and had cedar pollen-specific IgE antibodies (hereafter cedar antibodies) measured by Fluorescent Enzyme Immunoassay (FEIA) and the subjects were those who tested positive for cedar pollen-specific IgE antibodies (hereafter referred to as cedar antibodies) at 3.5 UA/mL or higher. Patients with severe bronchial asthma were excluded from the study because standardized cedar pollen extract solution was contraindicated.

The SLIT drug (standardized cedar pollen extract stock solution, 2000 JAU/mL bottle, Torii Pharmaceuticals, Tokyo, Japan) alone was administered to group S. The LEX (Lactobacillus acidophilus extract, B&S Corporation, Tokyo, Japan) alone was administered to group L. The SL group consisted of three patients in the S group, seven patients in the L group, and five patients in the SL group. One patient in the S group dropped out, two patients in the L group dropped out, and one patient in the SL group dropped out. Eleven patients in total were followed for three years. The follow-up was conducted for three pollen dispersal seasons from before the start of treatment in October 2015 to the end of pollen dispersal in May 2018. In Japan, cedar pollen dispersal usually begins around early January. Therefore, treatment was started by late November at the latest. 2000 JAU/day of SLIT drug was administered as a maintenance dose after a two-week dose increase period according to the dosing schedule in the package insert. LEX intake was two packets (10 mL x 2) per day; the SL group started LEX intake at the same time as SLIT, and the L group started LEX during November in conjunction with the start of SLIT.

As the primary outcome endpoint, patients were examined during the pollen dispersal period (mid-February to mid-March) in the first, second, and third years of treatment, and the severity classification of allergic rhinitis symptoms (severity score) was recorded. Swelling of the mucosa of the inferior turbinate, color tone of the mucosa of the inferior turbinate, amount of aqueous secretion, and the nature of nasal discharge were the examination items, each of which was rated on a 5-point scale from 0 to 4, and the total score of the four items was used as the severity score. In addition, subjective symptoms were surveyed using the Japanese Standard QOL Questionnaire for Allergic Rhinitis (JRQLQ No. 1), and the total score of all items was used as the QOL score [14]. Secondary outcome measures included nonspecific IgE (radioimmunosorbent test: IgE-RIST) test before the start of treatment (before pollen dispersal) and during the pollen dispersal period (mid-February to mid-March) in the first, second and third years of treatment, cedar-specific IgE ( (radioallergosorbent test: IgE-RAST) test. The above antibody tests were performed at Showa Medical Science Co. The values during the pollen dispersal period (mid-February to mid-March) were divided by the values before the start of treatment (before the pollen dispersal), and the IgE-RAST increase rate was calculated and compared.

Statistical treatment was performed using the Friedman test for changes over time within groups in severity and QOL evaluation scores of allergic rhinitis symptoms, IgE-RIST values, and IgE-RAST values, and the Kruskal-Wallis test for between-group comparisons and IgE-RAST increase rates for each year. Multiple comparisons were made by Scheffe's multiple comparison test. Outliers were detected using the Smirnoff-Grubbs test for initial IgE-RIST and IgE-RAST values. Spearman's rank correlation analysis was used to correlate severity scores and QOL scores. Statistical analysis was performed using Excel Statistics (version 3.21, Social Information Service Co., Ltd.), and a risk rate of less than 5% was judged to be statistically significant.

This study was conducted with the approval of the Ethical Review Committee of Rex Labs, a nonprofit corporation (certification number 002). Oral and written informed consent was obtained from all study participants, and the study was conducted in compliance with the Declaration of Helsinki. When handling the data and other materials related to the study, we gave sufficient consideration to the protection of the confidentiality of the subjects and did not include any information that could identify the subjects when we published the results of the study.

## Results

**Figure 1:**
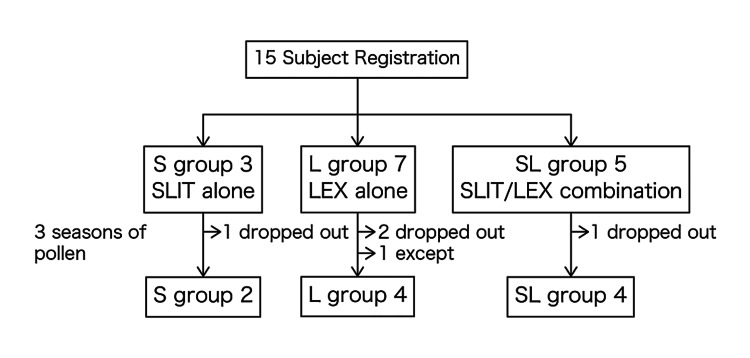
Subject flow This figure depicts patient enrollment by group, including drop-outs. We used the Smirnoff-Grubbs test to analyze pre-treatment IgE-RIST and cedar antibody levels and patients with high baseline antibody levels were excluded. SLIT: sublingual immunotherapy, LEX: Lactobacillus acidophilus extract, RIST: radio immunosorbence test, S: standardized cedar pollen extract group, L: lactobacillus-generated extract group, SL: standardized cedar pollen extract and lactobacillus-generated extract combination group

Figure 1 shows the subject flow. Fifteen subjects were included in the study, but four subjects withdrew during the study. One subject in group L had higher IgE-RIST and IgE-RAST (IgE-RIST: 1204 IU/ml, IgE-RAST: 100 UA/mL) before the start of treatment (before pollen dispersal) than the other subjects, and was excluded after outlier testing. As a result, the analysis included two subjects in the S group (one male, one female, mean age 38.0 years), four subjects in the L group (four females, mean age 29.8 years), and four subjects in the SL group (one male, three females, mean age 42.3 years).

**Figure 2:**
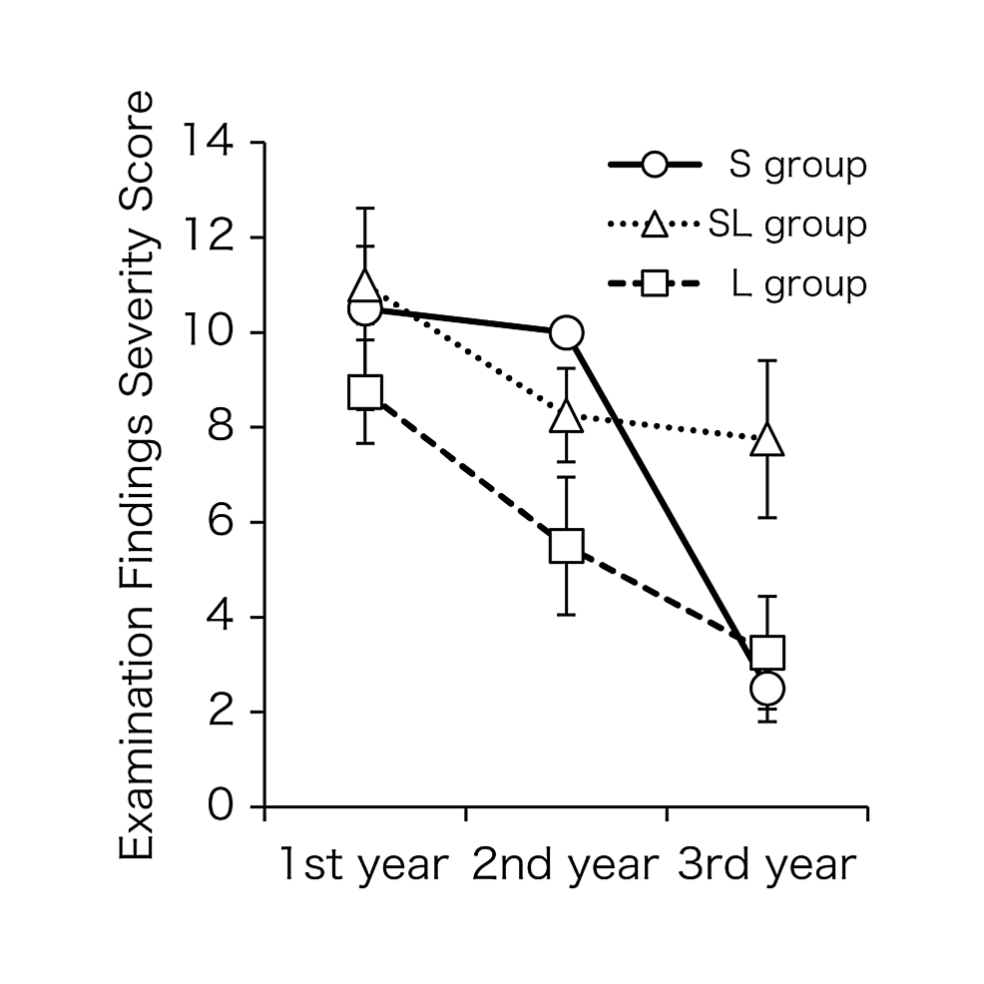
Severity score trends. All groups demonstrated improved severity ratings over time. Values of ○, △, and □ indicate mean score values, and error bars indicate standard deviations. S group (○): SLIT group, SL group (△): SLIT/LEX combination group, L group (□): LEX group. SLIT: sublingual immunotherapy, LEX: Lactobacillus acidophilus extract

Figure 2 shows changes in symptom severity over time and during the pollen dispersal periods. All groups experienced improved symptoms over time, with the L and SL groups showing near-significant improvement by Year 3 (p < 0.1). Although the number of patients in the S group was too small to draw statistical inferences, both groups improved significantly by Year 3 after starting treatment. There were no significant differences in severity scores between groups in each year.

**Figure 3:**
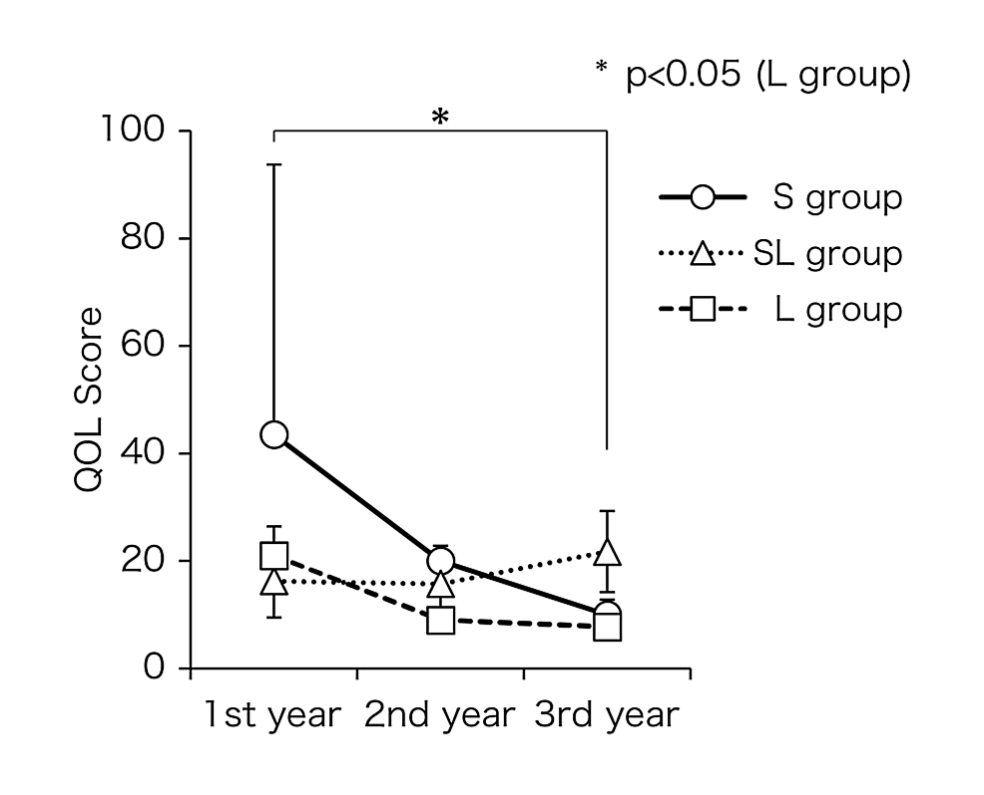
Change in QOL over time. The L and SL groups improved in Year 3. The L group showed significant improvement. ○, △, and □ indicate mean values, and error bars indicate standard deviations. S group (○): SLIT group, SL group (△): SLIT/LEX combination group, L group (□): LEX group. * p<0.05. Friedman test p-values are included in the figure above. QOL: quality of life, SLIT: sublingual immunotherapy, LEX: Lactobacillus acidophilus extract

**Figure 4:**
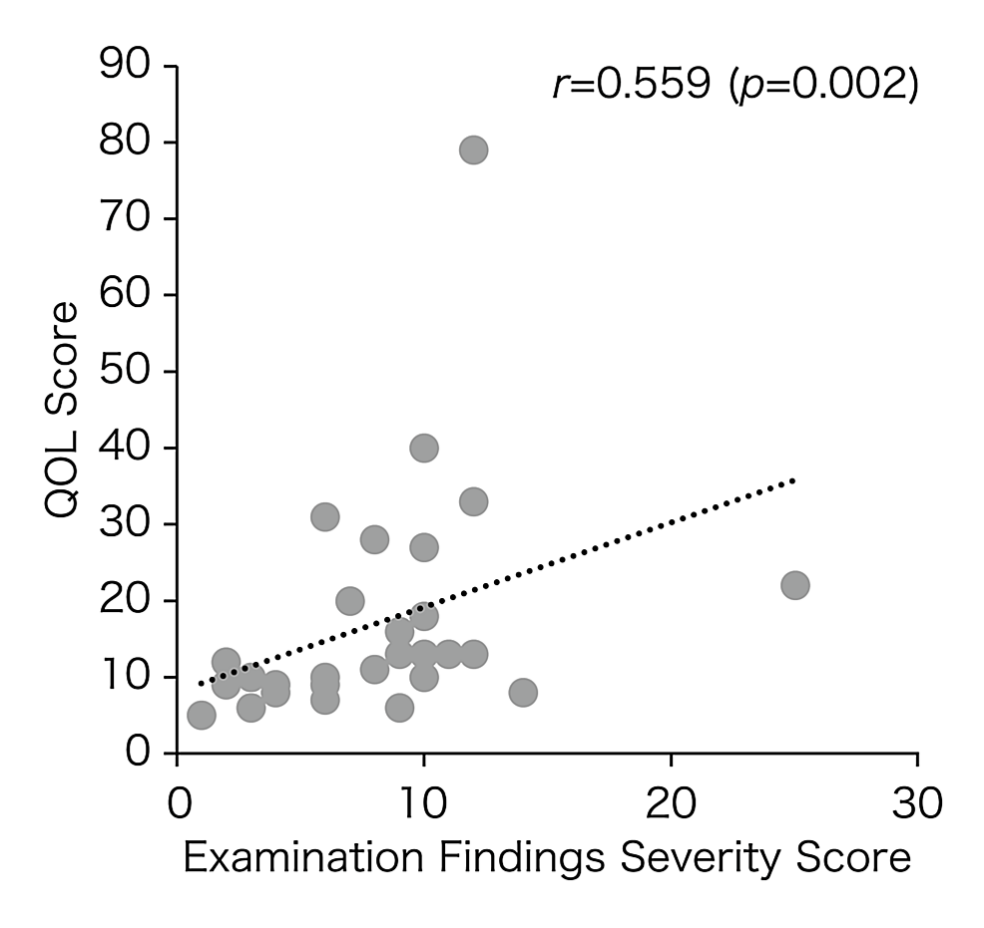
Correlation between severity and QOL scores in patients with cedar pollen allergies. A significant positive correlation is observed. Values in the graph show Spearman's rank correlation coefficient (r). Numbers in parentheses indicate p-values. S group: SLIT group, SL group: SLIT/LEX combination group, L group: LEX group. QOL: quality of life, SLIT: sublingual immunotherapy, LEX: Lactobacillus acidophilus extract

Figure 3 presents the groups’ QOL changes during the pollen dispersal period. The QOL scores of the S and L groups improved over time during treatment Years 1-3. Meanwhile, the L and SL groups tended to have lower QOL scores in Year 1. Group L showed a statistically significant improvement (p < 0.05) between treatment Years 1 and 3. Figure 4 shows the correlation between severity and QOL scores; note the positive, moderately strong correlation (r = 0.559, p = 0.002).

Table 1 shows the results of antibody tests. The IgE-RIST before the start of treatment (before pollen dispersal) was greater in the L, SL, and S groups, in that order, but there were no significant differences among the groups; in the S group, an increasing trend was observed in the IgE-RIST during the pollen dispersal period in the first year of treatment, but no significant changes were observed in the SL and L groups during the study period. In the S and SL groups, IgE-RAST showed an increasing trend in the first year of treatment, returned to the same level as before the start of treatment (before pollen dispersal) in the second year, and showed a decreasing trend in the third year. No significant changes were observed in intra-group comparisons. In the L group, a decreasing trend was observed from the first year of treatment, and the values showed a decreasing trend throughout the treatment period. In the second and third years of treatment, there was a significant (p<0.05) decrease compared to that before the start of treatment (before pollen dispersal).

**Table 1:** Changes in IgE-RIST and cedar pollen-specific IgE levels There was no significant difference between the S group, SL group and L group in IgE-RIST and IgE-RAST values. No significant difference was observed in the IgE-RIST values when comparing changes over time within each group. The IgE-RAST level in the L group decreased significantly (p<0.05) in the second and third years after the start of treatment compared to before the start of treatment (before pollen dispersal). Mean ± standard deviation. * p<0.05 RIST: radio immunosorbence test, RAST: radioallergosorbent test

item	Group	Before pollen dispersal	Pollen dispersal period		
			1st year	2nd year	3rd year
IgE-RIST (IU/mL)	S group	41.1±9.8	149.5±68.2	64.9±8.2	55.4±8.5
	SL group	206.6±76.0	257.7±99.0	204..7±60.5	203.4±109.2
	L group	316.5±142.4	329.1±159.9	291.7±129.4	304.4±150.9
Cedar-specific IgE (UA/mL)	S group	7.6±1.4	68.7±25.6	24.4±6.0	12.2±2.9
	SL group	33.6±12.0	54.0±24.3	35.3±18.8	20.4±9.7
	L group	28.0±9.9	19.2±11.5	15.3±8.4 ＊	15.6±9.0 ＊

## Discussion

This study compared the efficacy of SLIT and LEX separately in a small number of cedar pollinosis patients. We also compared the effects of SLIT and LEX in combination. Although a sufficient number of subjects could not be obtained because some patients withdrew during the course of the study, the results of the comparison of the three groups (SLIT group, LEX group, and SLIT and LEX combined group) showed that QOL scores and IgE-RAST values decreased significantly in the LEX group, suggesting that LEX is useful in treating cedar pollinosis.

Cedar pollen dispersal in Japan in 2016, the first year of treatment, and 2017, the second year of treatment, was similar to normal, but in 2018, the third year of treatment, a large dispersal was observed. Even in the third year of treatment, the year of heavy dusting, each group showed improvement in severity and QOL scores, the primary outcome measures, and in IgE-RIST and IgE-RAST, the secondary outcome measures, suggesting that the treatment in each group was effective. Some degree of correlation was also observed between severity score and QOL score, suggesting that there was a correlation between subjective symptoms and other examination findings. Quality of life itself was considered to have improved along with the improvement of actual symptoms.

SLIT is reported to be gradually effective over a period of three years [15], and the S group in this study also required three years of treatment. However, about 20% of patients do not respond to SLIT and are considered nonresponders [16]. The difference between effective and ineffective cases and the difference in the duration of efficacy after the completion of treatment are still unclear. This suggests that the LEX intake groups (SL and L groups) may show an early onset of effect.

One problem with the study design of this study is that the severity and quality of life score assessment and testing for antibodies during the pollen dispersal period (2015) prior to treatment were not available. The reason is that patients do not visit clinics without the appearance of allergic symptoms and visit clinics only after the appearance of allergic symptoms. Patients who visit the clinic prophylactically before the appearance of allergic symptoms are rare, and it was not possible to select subjects who had not yet developed allergic symptoms. As a result, this study only had data after the start of treatment. However, considering the trend that severity and QOL scores improved with each passing year in the second and third years compared to the first year of treatment, it is considered that both study groups improved against cedar pollinosis. These results suggest that LEX prescriptions (SL and L groups) may improve QOL scores already in the first year of treatment, and that QOL scores may improve earlier than in the S group. LEX alone or in combination with SLIT is effective for early onset of treatment effect with improvement of QOL in patients with cedar pollinosis.

Early selection of effective SLIT patients is considered necessary to reduce patient burden and increase motivation for treatment. Although the effectiveness of SLIT is often judged based on severity and QOL scores, objective evaluation based on biomarkers, rather than subjective symptoms alone, is considered necessary to determine the effectiveness of SLIT. However, objective evaluation using biomarkers, rather than subjective symptoms alone, is considered to be necessary [17]. In this study, changes in IgE-RIST and IgE-RAST were also investigated. The results showed a transient increase in cedar antibody levels in the first year of treatment in the S and SL groups. The increase was suppressed in the second and third years. On the other hand, the L group did not show a transient increase in cedar antibody levels in the first year, but showed a decreasing trend with each passing year, and the levels remained lower during the pollen dispersal season than during the period when cedar pollen was not dispersed. The SL group showed a lower transient increase in IgE-RAST in the first year than the S group, suggesting that ingestion of LEX has the effect of suppressing the increase in IgE-RAST during the pollen dispersal period; IgE-RAST may be a biomarker that can evaluate cedar pollinosis.

Oral administration of LEX in mice has been reported to increase natural killer T (NKT) cells and induce production of interferon-γ in mucosal immunity of the digestive tract [11]. In other words, LEX is thought to activate the innate immune system and regulate the immune balance between type 1 helper T cells (Th1) and type 2 helper T cells (Th2). It is also thought to be involved in the clinical improvement effect of LEX on cedar pollinosis and reduction of cedar pollen-specific antibody production in this study. Lactobacillus acidophilus as a probiotic is also thought to alleviate type I allergy, including cedar pollinosis, by regulating the balance of effector cells such as Th1, Th2, or Th17 cells [6,18]. In allergen immunotherapy, antigens are administered at higher concentrations than normally exposed, and are thought to suppress allergic reactions by locally inducing Th1 cells [19]. It has also been noted that fluctuations in pathogenic Th2 subsets in allergen immunotherapy are also important for therapeutic efficacy [20].

The combination of SLIT and LEX may lead to an early onset of SLIT efficacy through the additive effects of LEX's activation of Th1 cells via the innate immune system and SLIT's local induction of Th1 cells and specific Th2 cell fluctuations. However, the results of this study did not reveal any benefit of the combination therapy of LIT and LEX over SLIT alone, suggesting that LEX has a possible mechanism of action through the intestinal immune system, such as regulation of intestinal permeability, normalization of host intestinal microbiota, enhancement of intestinal immune barrier function, and regulation of inflammatory and anti-inflammatory cytokine balance. It is possible that the superiority of combination therapy with SLIT and LEX was not clear because the mechanism of action of SLIT is different from that of LEX, which induces Th1-type immune responses and mitigates Th2-type immune responses via the innate immune system and the acquired immune system. However, considering that there were no invalid cases in the SLIT/LEX combination treatment group in this study, it is possible that the combination of SLIT and LEX may have acted as a salvage therapy to reduce the number of invalid cases.

At present, SLIT is a therapy for only limited allergic symptoms such as dust mites and cedar pollen. However, it may be possible to apply SLIT to various allergic diseases by applying food-derived ingredients more widely. Patients with allergic rhinitis rarely have allergic reactions only to cedar pollen or mites, and it is a fact that many patients react to various allergens. The application of LEX and other food-derived ingredients is thought to contribute greatly to alleviating allergic symptoms and improving the quality of life of patients [21]. In addition, the use of food-derived ingredients may be useful as a fundamental solution to the problems of side effects caused by long-term administration of antiallergic drugs and inflammation suppressants, which are frequently used in the treatment of allergic diseases, as well as the problems of being only a symptomatic treatment.

This study was a pilot study on a small scale due to the long observation period and the large number of dropout cases, making it difficult to obtain eligible cases. the combined effect of LEX and SLIT is considered useful, but the number of cases needs to be increased in the future in order to determine statistically significant differences. Based on the results of this study, we would like to increase the number of cases and proceed with the study by specifying future issues and research methods.

## Conclusions

This study examined the usefulness of LEX as a treatment for cedar pollinosis. We also examined whether the combination of SLIT and LEX could have an early onset of therapeutic effect on cedar pollinosis. We also examined whether LEX could be useful as a salvage therapy for patients with inadequate response to treatment.

The severity score and nonspecific IgE levels were not significantly different among the three groups, and the quality of life score decreased significantly between the first and third years of treatment in the L group. In the L group, cedar pollen-specific IgE levels decreased significantly in the second and third years of treatment. Based on the severity and quality of life scores, it was expected that the S and SL groups would require three years of treatment before the onset of efficacy, while the L group showed a trend toward improvement in quality of life scores and cedar pollen-specific IgE levels from the first year of treatment, suggesting that an early onset of efficacy can be expected for LEX. and LEX was not clear.

Since LEX is expected to have an early onset of effect, it may be useful as a salvage therapy to reduce the incidence of ineffective cases by combining LEX intake from the early stage of treatment. There is little evidence on the immunomodulatory effects of postbiotic components in humans, and there are no studies within the authors' reach that have evaluated the combination therapy of SLIT and LEX. This study is reported here as a potential source of evidence.
